# Supplementary material for: Polymer-Nanodiscs as a Novel Alignment Medium for High-Resolution NMR-Based Structural Studies of Nucleic Acids
Source: Biomolecules. 2022 Nov 3;12(11):1628. doi: 10.3390/biom12111628 (PMC9687133; doi:10.3390/biom12111628)
Supplement: Supplementary file 1 [file biomolecules-12-01628-s001.zip › biomolecules-1969679-supplementary.pdf]

Supplementary Information

Polymer-nanodiscs as a novel alignment medium for high-resolution NMR-based structural studies of nucleic acids

Bankala Krishnarjuna,[1#] Thirupathi Ravula,[1,2#] Edgar M. Faison,[3#] Marco Tonelli,[2] Qi Zhang,[3*] and Ayyalusamy Ramamoorthy[1*]

[1]Biophysics Program, Department of Chemistry, Biomedical Engineering, and Macromolecular Science and Engineering, and Michigan Neuroscience Institute, University of Michigan, Ann Arbor, MI 48109, USA.

[2]National Magnetic Resonance Facility at Madison, University of Wisconsin-Madison, Madison, WI 53706, USA.

[3]Department of Biochemistry and Biophysics, University of North Carolina at Chapel Hill, Chapel Hill, NC 27599, USA.

*****Correspondence: zhangqi@unc.edu; ramamoor@umich.edu

[#]Equal contribution to this work.

Table S1. List of 2D ARTSY-HSQC NMR experiments performed on RNA in magnetically-aligned polymer-nanodiscs.

NMR experiment	Time-domain data size (complex points)		Offset (ppm)		Spectral width (ppm)		Number of scans	ARTSY delay (ms)
	t_1	t_2	F_1	F_2	F_1	F_2		
^{1}H-^{15}N-BEST-HSQC-ARTSY	1024	128	4.868 (^{1}H)	152.73 (^{15}N)	25.1 (^{1}H)	24.67 (^{15}N)	40	11.5
^{1}H-^{13}C-HSQC-ARTSY Aromatic	1024	220	4.868 (^{1}H)	146.131 (^{13}C)	16.67 (^{1}H)	26.51 (^{13}C)	40	5.0
^{1}H-^{13}C-HSQC-ARTSY H5/H1’	1024	220	4.868 (^{1}H)	97.657 (^{13}C)	16.67 (^{1}H)	26.51 (^{13}C)	40	6.0
^{1}H-^{13}C-HSQC-ARTSY Constant-Time Ribose	1024	202	4.868 (^{1}H)	84.772 (^{13}C)	16.67 (^{1}H)	47.06 (^{13}C)	48	6.5

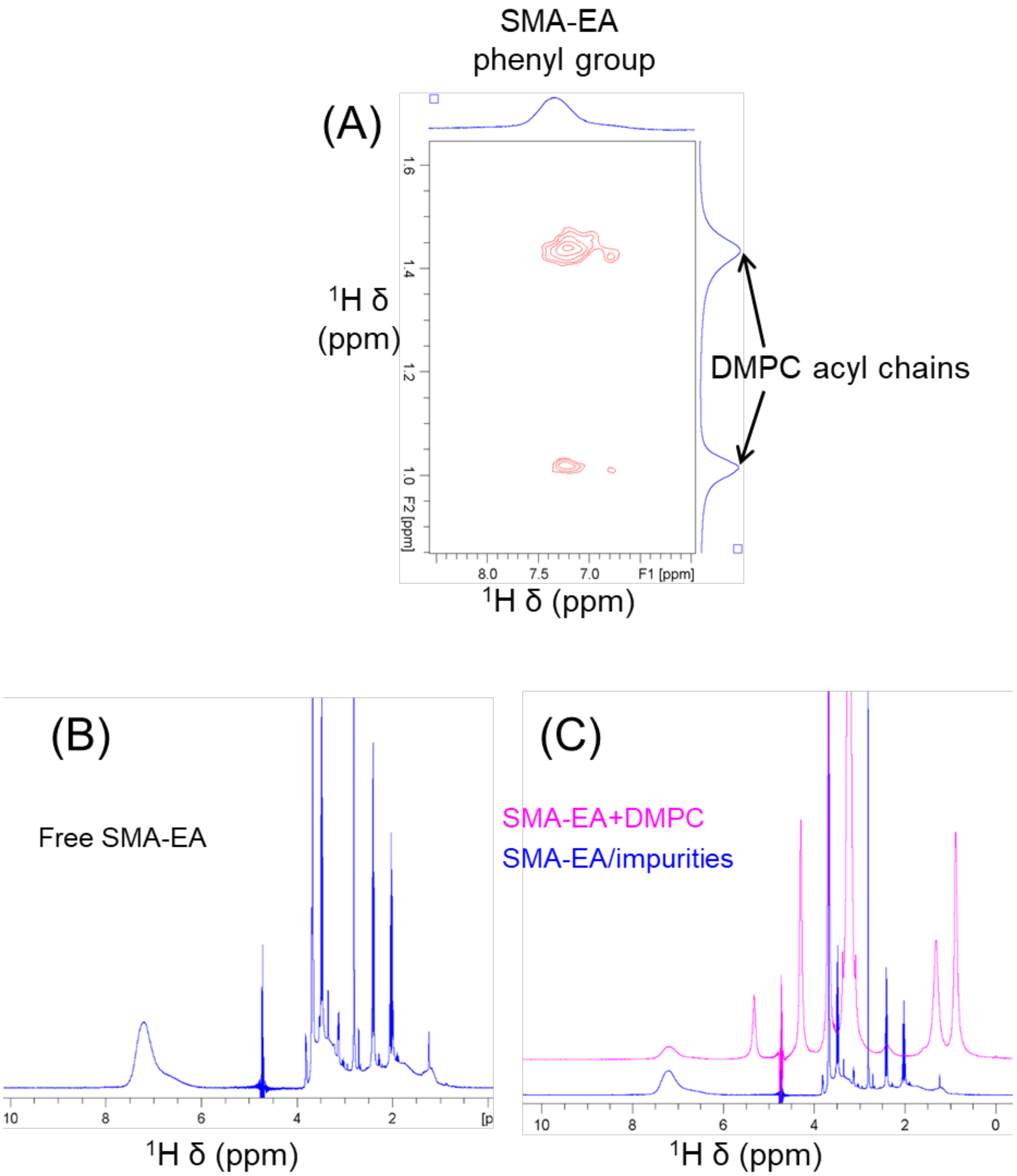

Figure S1. (**A**) Partial 2D ^{1}H/^{1}H NOESY spectrum (with 1D ^{1}H projections) of the SEC-purified SMA-EA DMPC-nanodiscs before concentrating the sample for RDC measurements. The NOE cross-peaks between protons of the DMPC acyl chain and the SMA-EA phenyl group, indicating the polymer-lipid interactions in nanodiscs. The spectrum was recorded using a room-temperature TXI-probe operated at 308 K. (**B**) ^{1}H NMR spectrum of the free SMA-EA polymer in SEC fractions eluted between 14-20.5 mL (See Figure 1B in the main text). The absence of methyl ^{1}H peaks confirms the absence of DMPC lipids in these fractions. (**C**) Overlay of ^{1}H NMR spectra of SMA-EA DMPC-nanodiscs (magenta) and free SMA-EA polymer (blue) (see the SEC profile in Figure 1B).

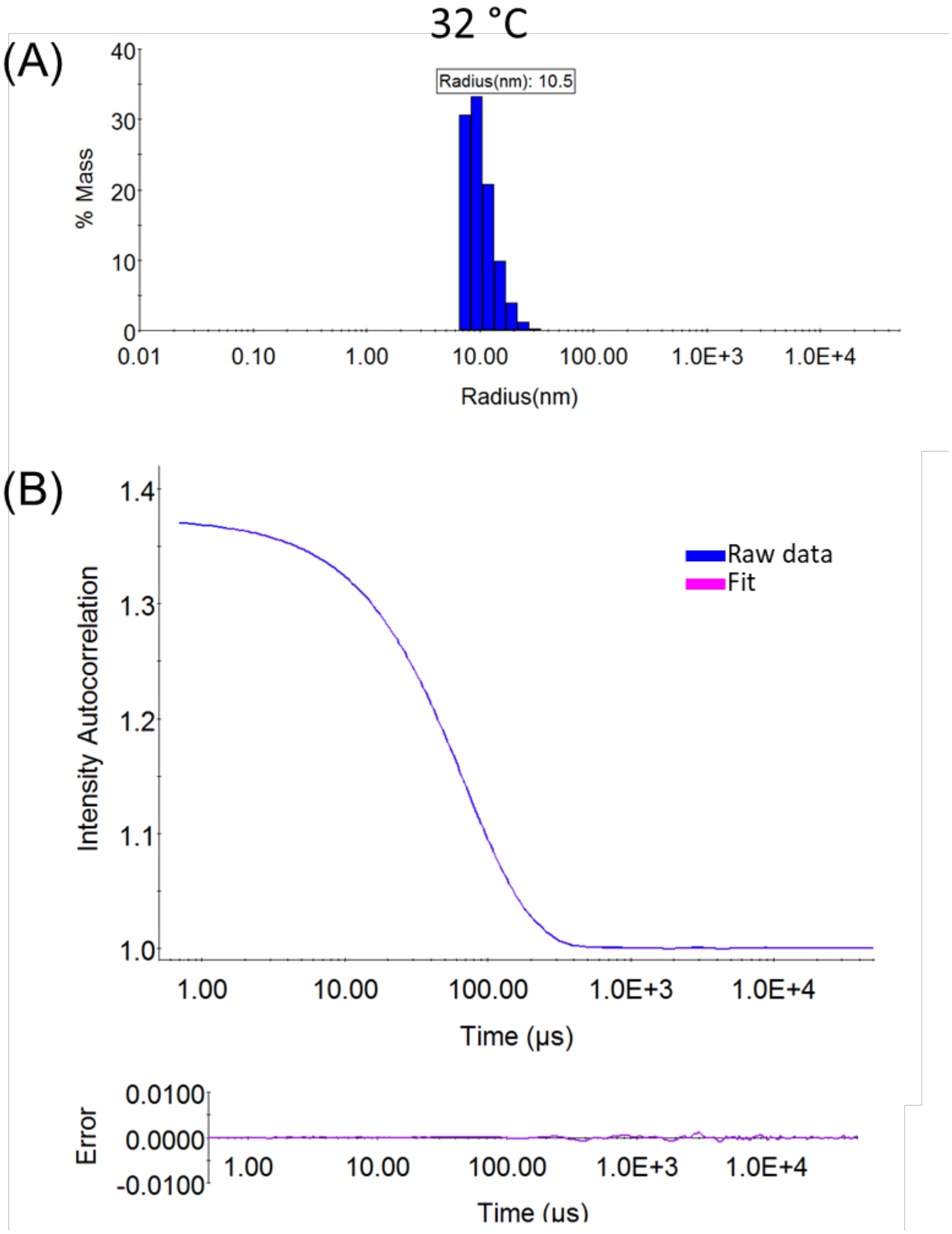

Figure S2. (**A**) Dynamic light scattering profile of polymer-nanodiscs (1:1 w/w ratio of SMA-EA:DMPC). As indicated, the estimated hydrodynamic radius of polymer-nanodiscs at 32 °C was 10.5 nm. (**B**) The autocorrelation function used to estimate the hydrodynamic radius and fits are shown; residuals of the fitting are shown at the bottom.

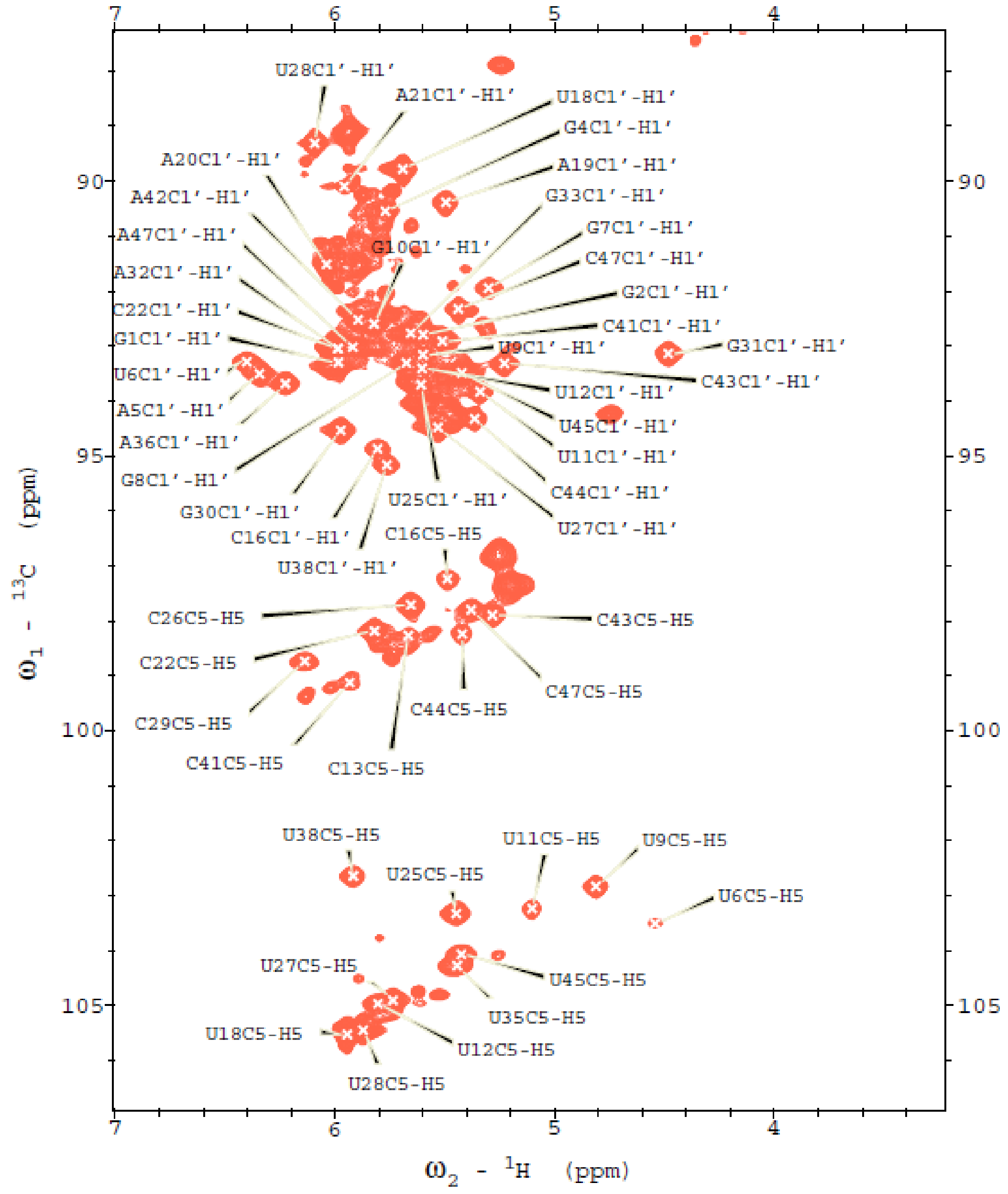

U28C1′-H1′
A21C1′-H1′
U18C1′-H1′
G4C1′-H1′
A19C1′-H1′
A20C1′-H1′
A42C1′-H1′
G33C1′-H1′
A47C1′-H1′
G7C1′-H1′
G10C1′-H1′
C47C1′-H1′
A32C1′-H1′
G2C1′-H1′
C22C1′-H1′
C41C1′-H1′
G1C1′-H1′
U9C1′-H1′
G31C1′-H1′
U6C1′-H1′
C43C1′-H1′
U12C1′-H1′
A5C1′-H1′
U45C1′-H1′
A36C1′-H1′
U11C1′-H1′
G8C1′-H1′
C44C1′-H1′
U25C1′-H1′
G30C1′-H1′
U27C1′-H1′
C16C1′-H1′
C16C5-H5
U38C1′-H1′
C26C5-H5
C43C5-H5
C22C5-H5
C47C5-H5
C44C5-H5
C29C5-H5
C41C5-H5
C13C5-H5
U38C5-H5
U11C5-H5
U9C5-H5
U25C5-H5
U6C5-H5
U27C5-H5
U45C5-H5
U35C5-H5
U18C5-H5
U12C5-H5
U28C5-H5
7
6
5
4
90
95
100
105
ω_1 - ^{13}C (ppm)
ω_2 - ^{1}H (ppm)

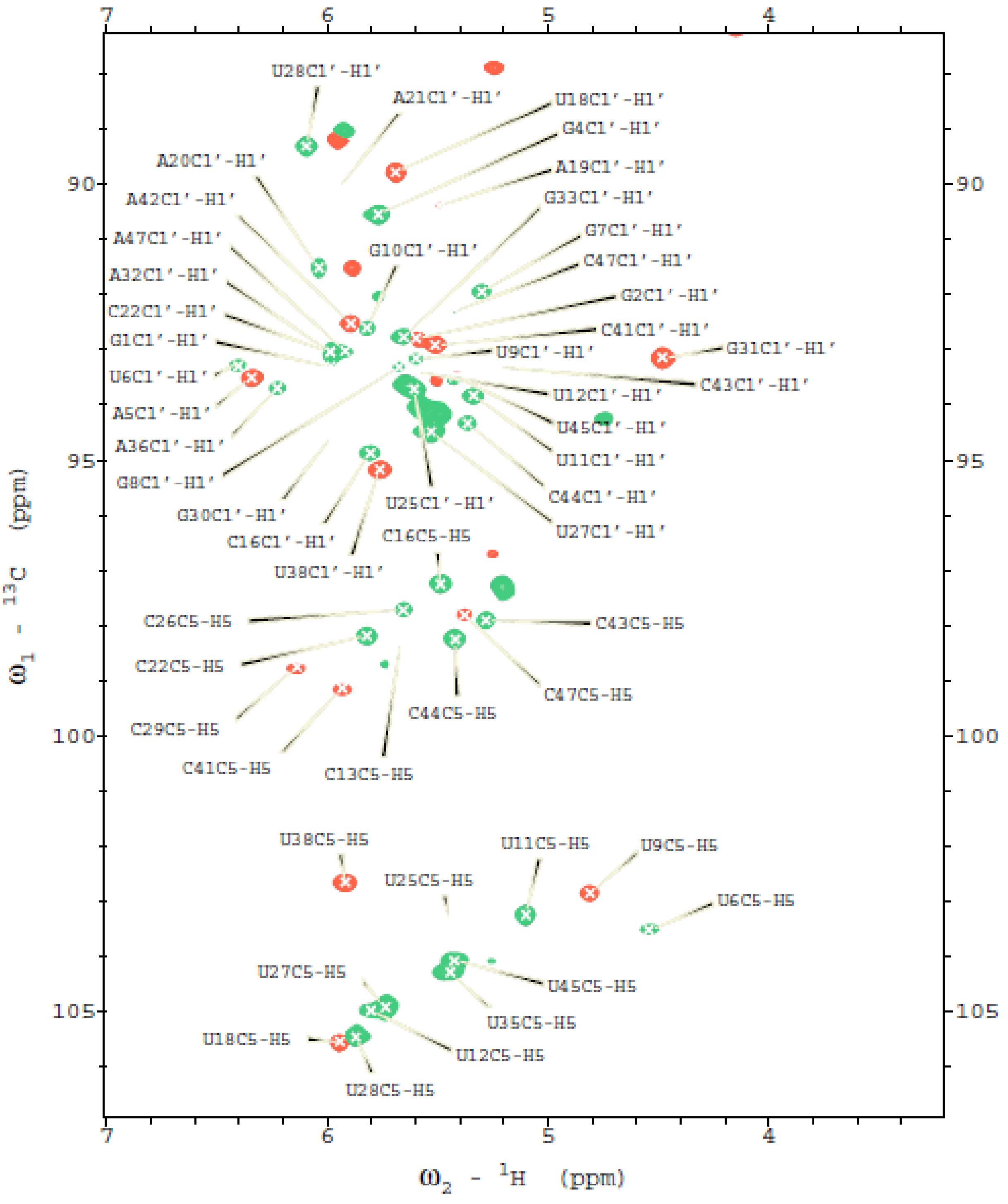

7
6
5
4
90
95
100
105
U28C1'-H1'
A21C1'-H1'
U18C1'-H1'
G4C1'-H1'
A20C1'-H1'
A19C1'-H1'
A42C1'-H1'
G33C1'-H1'
G7C1'-H1'
A47C1'-H1'
G10C1'-H1'
C47C1'-H1'
A32C1'-H1'
G2C1'-H1'
C22C1'-H1'
C41C1'-H1'
G1C1'-H1'
G31C1'-H1'
U9C1'-H1'
U6C1'-H1'
C43C1'-H1'
U12C1'-H1'
A5C1'-H1'
U45C1'-H1'
A36C1'-H1'
U11C1'-H1'
G8C1'-H1'
C44C1'-H1'
U25C1'-H1'
G30C1'-H1'
U27C1'-H1'
C16C1'-H1'
C16C5-H5
U38C1'-H1'
C26C5-H5
C43C5-H5
C22C5-H5
C47C5-H5
C44C5-H5
C29C5-H5
C41C5-H5
C13C5-H5
U38C5-H5
U11C5-H5
U9C5-H5
U25C5-H5
U6C5-H5
U27C5-H5
U45C5-H5
U35C5-H5
U18C5-H5
U12C5-H5
U28C5-H5
ω1 - 13C (ppm)
ω2 - 1H (ppm)

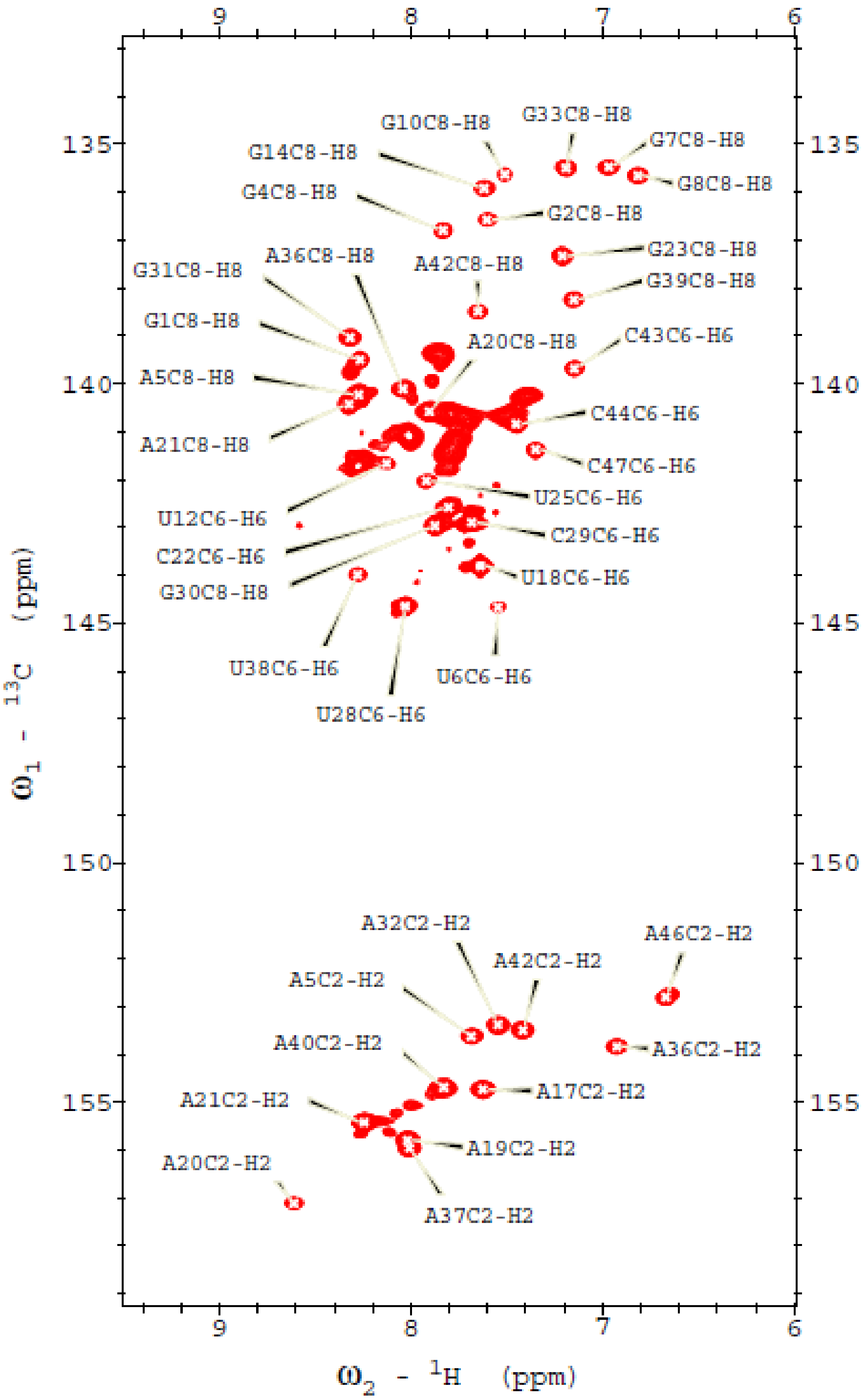

G10C8-H8
G33C8-H8
G7C8-H8
G14C8-H8
G8C8-H8
G4C8-H8
G2C8-H8
A36C8-H8
G23C8-H8
G31C8-H8
A42C8-H8
G39C8-H8
G1C8-H8
A20C8-H8
C43C6-H6
A5C8-H8
C44C6-H6
A21C8-H8
C47C6-H6
U25C6-H6
U12C6-H6
C29C6-H6
C22C6-H6
G30C8-H8
U18C6-H6
U38C6-H6
U6C6-H6
U28C6-H6
A32C2-H2
A46C2-H2
A42C2-H2
A5C2-H2
A40C2-H2
A36C2-H2
A17C2-H2
A21C2-H2
A19C2-H2
A20C2-H2
A37C2-H2
9
8
7
6
135
140
145
150
155
ω1 - 13C (ppm)
ω2 - 1H (ppm)

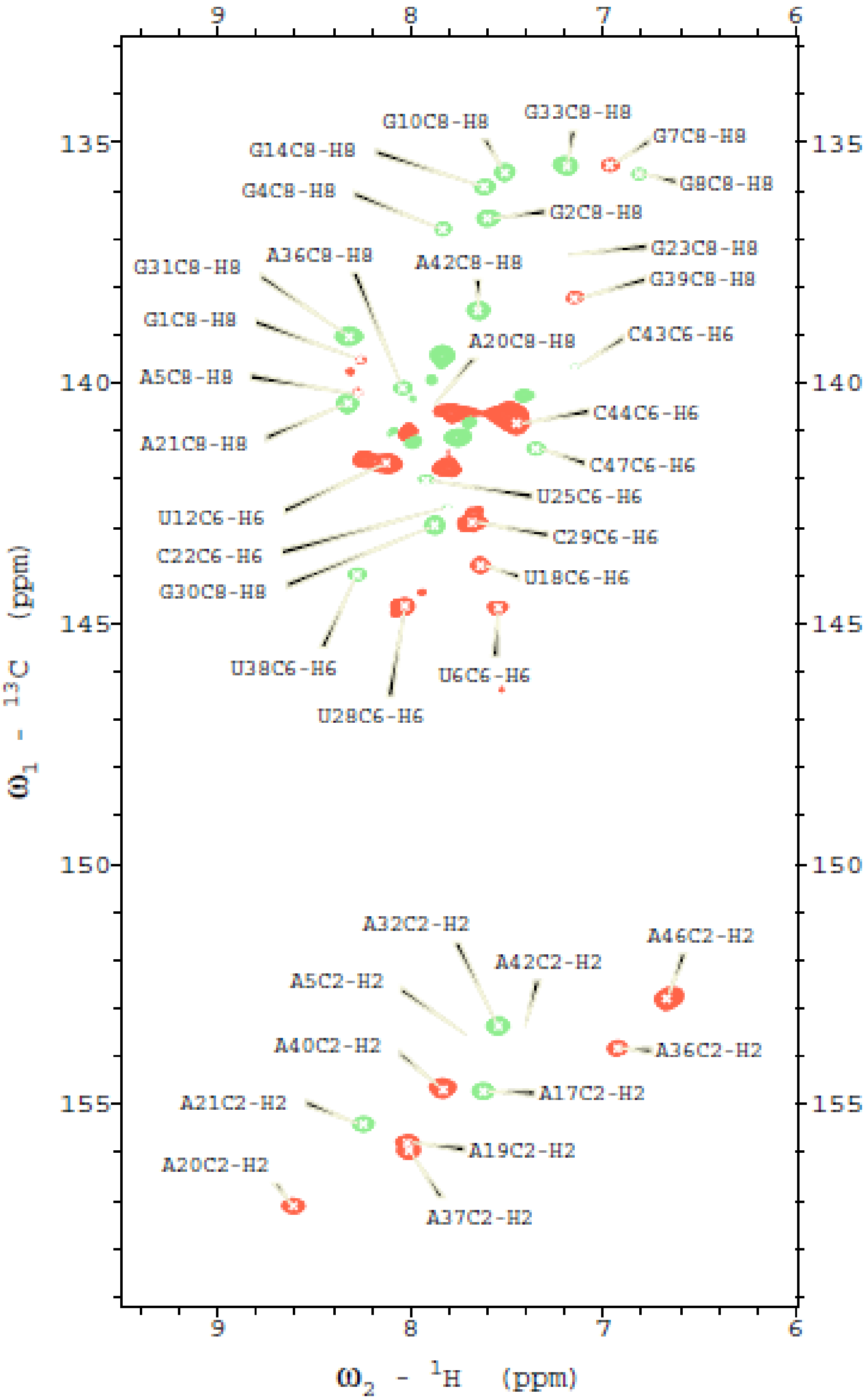

Figure S3. 2D ARTSY NMR spectra of fluoride riboswitch aptamer in magnetically-aligned SMA-EA DMPC-nanodiscs. The spectra were acquired on a Varian VNMRS 600 MHz NMR spectrometer operated with the probe temperature of 308 K. The spectra were processed using NMRPipe and analyzed using NMRFARM Sparky with previously reported NMR assignments for the fluoride-bound state.[1]

(A)

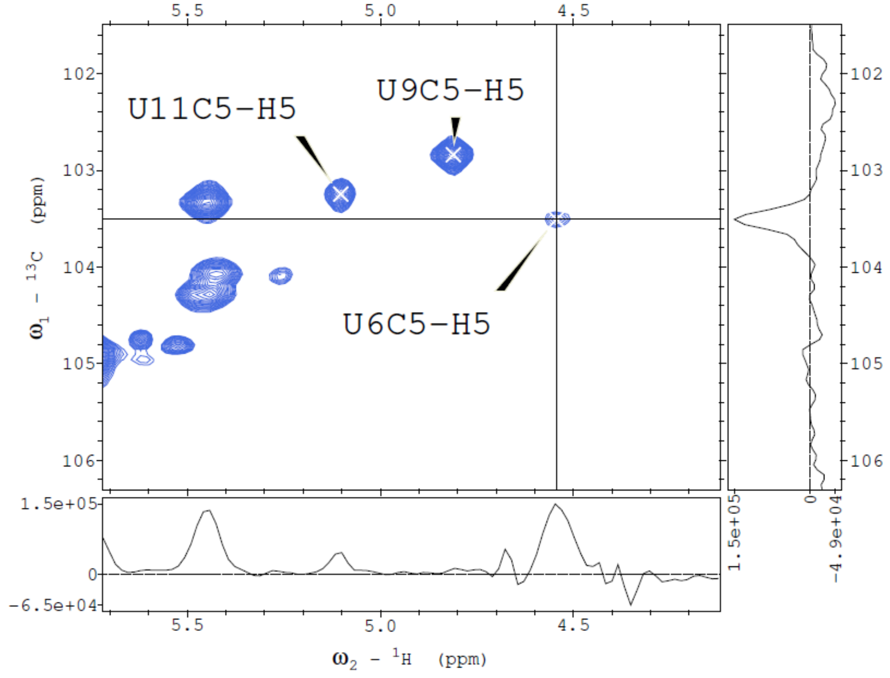
U11C5–H5
U9C5–H5
U6C5–H5
ω1 – 13C (ppm)
ω2 – 1H (ppm)

(B)

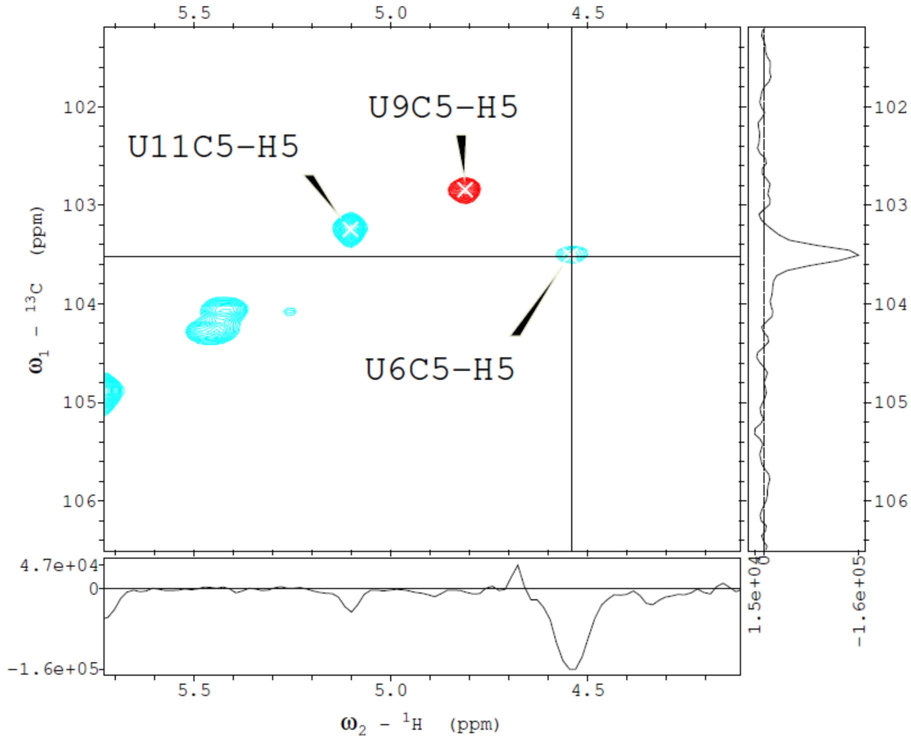
U11C5–H5
U9C5–H5
U6C5–H5
ω1 – 13C (ppm)
ω2 – 1H (ppm)

(C)

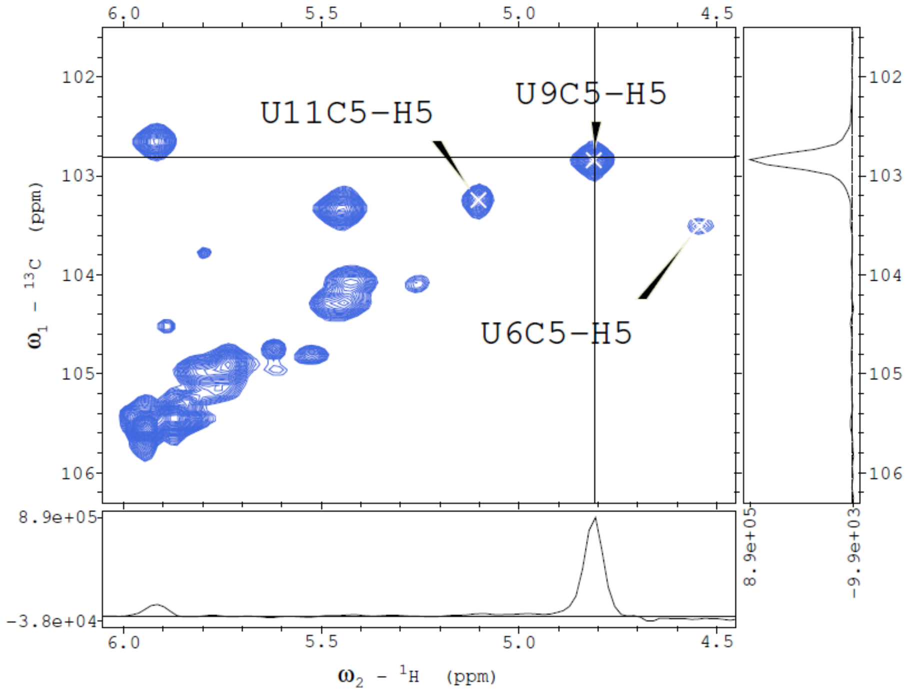
U11C5-H5
U9C5-H5
U6C5-H5
6.0
5.5
5.0
4.5
102
103
104
105
106
8.9e+05
-3.8e+04
-9.9e+03
ω1 - 13C (ppm)
ω2 - 1H (ppm)

(D)

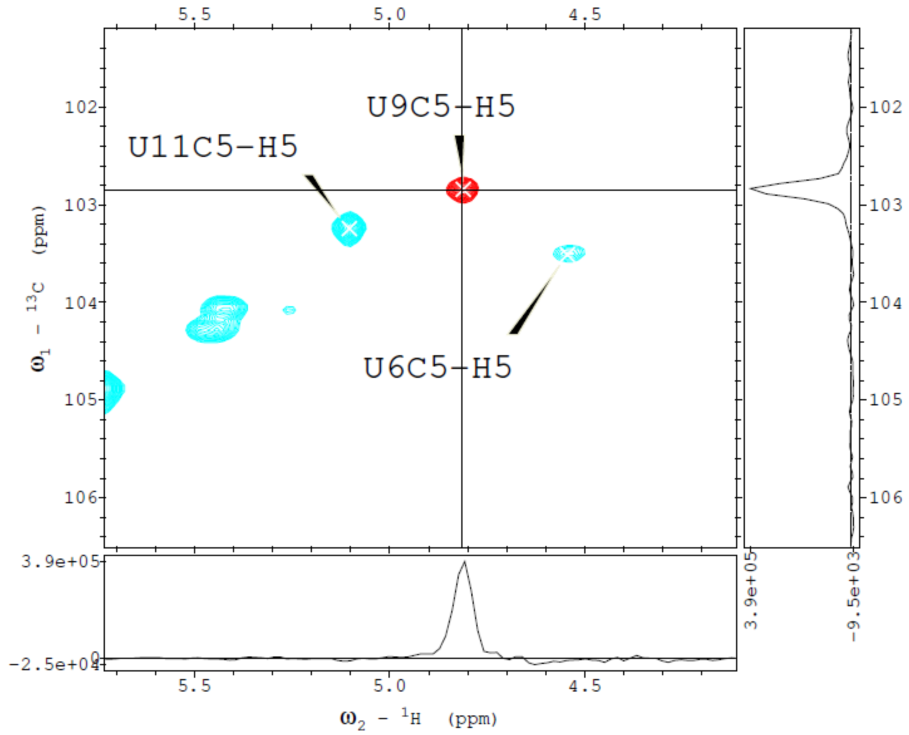
U9C5-H5
U11C5-H5
U6C5-H5
5.5
5.0
4.5
102
103
104
105
106
3.9e+05
-2.5e+04
0
-9.5e+03
ω1 - 13C (ppm)
ω2 - 1H (ppm)

(E)

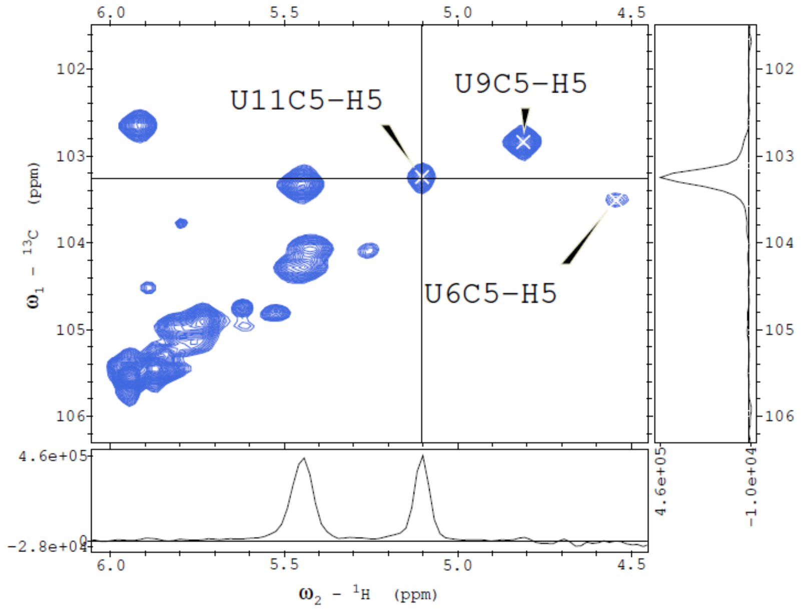

(F)

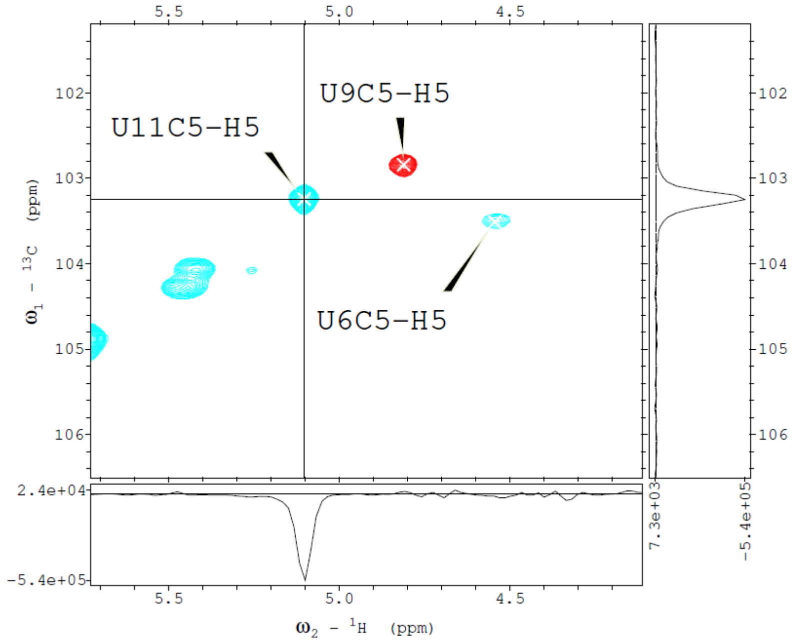

Figure S4. Different regions of 2D ARTSY reference (A, C, E) and the attenuated (B, D, F) spectra of the fluoride riboswitch aptamer in magnetically-aligned SMA-EA DMPC-nanodiscs showing 1D slices for the assigned cross-peaks. The cross-peaks with positive and negative phases in the attenuated spectra are colored red and cyan, respectively. The spectra were processed using NMRPipe and analyzed using NMRFARM Sparky with previously reported NMR assignments for the fluoride-bound state.[1]

References

(1) Zhao, B.; Guffy, S. L.; Williams, B.; Zhang, Q. An excited state underlies gene regulation of a transcriptional riboswitch. *Nat. Chem. Biol.* **2017**, *13*, 968-974.
